# Exercise Effects on Motor Skill Consolidation and Intermuscular Coherence Depend on Practice Schedule

**DOI:** 10.3390/brainsci12040436

**Published:** 2022-03-24

**Authors:** Ali Khan, Jyotpal Singh, J. Patrick Neary, Cameron S. Mang

**Affiliations:** Faculty of Kinesiology and Health Studies, University of Regina, 3737 Wascana Parkway, Regina, SK S4S 0A2, Canada; aak502@uregina.ca (A.K.); jyotpal.singh@uregina.ca (J.S.); patrick.neary@uregina.ca (J.P.N.)

**Keywords:** motor learning, exercise, electromyography, intermuscular coherence, contextual interference

## Abstract

Cardiorespiratory or aerobic exercise immediately after practice of an upper-extremity motor skill task can facilitate skill consolidation, as demonstrated by enhanced performances at 24 h and 7-day retention tests. The purpose of this study was to examine the effect of acute cardiorespiratory exercise on motor skill consolidation when skill practice involved low and high levels of contextual interference introduced through repetitive and interleaved practice schedules, respectively. Forty-eight young healthy adults were allocated to one of four groups who performed either repetitive or interleaved practice of a pinch grip motor sequence task, followed by either a period of seated rest or a bout of high-intensity interval cycling. At pre- and post-practice and 24 h and 7-day retention tests, we assessed motor skill performance and β-band (15–35 Hz) intermuscular coherence using surface electromyography (EMG) collected from the abductor pollicis brevis and first dorsal interosseous. At the 7-day retention test, off-line consolidation was enhanced in the cardiorespiratory exercise relative to the rest group, but only among individuals who performed interleaved motor skill practice (*p* = 0.02). Similarly, at the 7-day retention test, β-band intermuscular coherence increased to a greater extent in the exercise group than in the rest group for those who performed interleaved practice (*p* = 0.02). Under the present experimental conditions, cardiorespiratory exercise preferentially supported motor skill consolidation and change in intermuscular coherence when motor skill practice involved higher rather than lower levels of contextual interference.

## 1. Introduction

In the last decade, multiple studies have examined acute cardiorespiratory or aerobic exercise effects on motor learning. Substantial evidence now suggests that a single bout of cardiorespiratory exercise performed close in time to motor skill practice can enhance both motor skill acquisition [1,2] and consolidation [3,4,5,6]. Administering a bout of cardiorespiratory exercise before practicing a motor task has a larger influence on skill acquisition, whereas exercise following the motor task primarily influences consolidation [5]. Further research suggests that the magnitude of these effects depends on exercise intensity. For example, Thomas and colleagues [6] found that performing high-intensity interval cycling following upper extremity motor skill practice resulted in significantly larger offline gains in performance compared to low-intensity continuous cycling.

One of the main mechanistic hypotheses for acute cardiorespiratory exercise effects on motor skill consolidation suggests that an exercise-induced upregulation of neurochemicals (e.g., brain-derived neurotrophic factor, dopamine) facilitates long-term potentiation (LTP)-like processes that support learning and memory [7,8,9]. A related line of research has identified moderate- and high-intensity exercise effects on the activity of excitatory and inhibitory interneurons within the human primary motor cortex (M1) that are also linked to the induction of LTP [10,11,12,13]. In other work, acute cardiorespiratory exercise at low-, moderate-, and high-intensity was reported to increase the response to non-invasive brain stimulation techniques designed to induce neuroplasticity in human M1 [14].

Besides cardiorespiratory exercise, many other ‘post-practice’ strategies (e.g., non-invasive brain stimulation, sleep) to enhance motor learning, and, specifically, skill consolidation, have been investigated [15,16]. However, motor skill consolidation is not solely dependent on post-practice conditions; a large body of research demonstrates that it can also be influenced by conditions introduced during practice [17]. Specifically, how a practice session is organized can influence skill consolidation by introducing contextual interference [18]. Contextual interference refers to the interference that a learner experiences when practicing multiple skills or variations of a skill within a single practice session [19]. A practice session can be organized in a repetitive schedule, such that an individual repeatedly practices the same motor task before moving on to other motor tasks that will also be practiced in the session. This approach would be considered to provide very little contextual interference. Conversely, a practice session can be organized in an interleaved schedule, whereby an individual practices a set of motor tasks in an unpredictable order, avoiding the execution of the same task in consecutive trials [20]. This trial-to-trial change in task demands provides a large degree of contextual interference that places greater demands on motor planning and memory processes than repetitive practice. The contextual interference effect is a phenomenon that has been observed in which repetitive practice results in better initial performance than interleaved practice, but interleaved practice results in better consolidation of the skill as reflected by reports of enhanced offline gains in performance across long-term (i.e., >24 h) retention intervals [17,21].

The neural substrates of motor learning are widespread and dependent on the stage of learning [22]. Generally, motor learning involves a shift from a period of high cognitive demand with increased frontal brain region activity toward automatization with increased activity in solely motor regions of the brain (i.e., M1). Yet, the relative contributions of these brain regions to motor skill consolidation depend on practice and post-practice conditions. For example, evidence suggests that skill consolidation following a session of task practice with an interleaved schedule relies heavily on the dorsolateral prefrontal cortex for consolidation, whereas consolidation following repetitive practice relies more on M1 [23]. Considering the effects of acute exercise on the neural substrates of motor learning, Ostadan and colleagues [24] determined that exercise after practice of an implicit serial reaction time task resulted in increases in corticospinal excitability that were positively correlated to off-line gains in skill performance. Notably, post-practice changes in corticospinal excitability in a resting control group were not associated with off-line gains. Their results may suggest that exercise promoted short-term neuroplasticity in corticospinal pathways that contributed to skill consolidation [24].

The purpose of this study was to examine the effect of high-intensity interval cycling on motor skill consolidation when a skill is practiced with either a “repetitive” or “interleaved” schedule. We hypothesized that the coupling of an interleaved schedule with a post-practice bout of exercise would have an additive effect on motor skill consolidation (i.e., their independent effects would sum together). This hypothesis was based on our assumptions that:

(1) neurochemicals upregulated by acute exercise [7,8,9] may support motor learning processes engaged by both repetitive and interleaved practice,
(2) increased demand on motor planning and memory processes provided by contextual interference [17,18] may enhance motor learning regardless of post-practice conditions (i.e., exercise or rest), and
(3) exercise effects on learning-related changes in corticospinal excitability [24] and contextual interference links to dorsolateral prefrontal cortex activity [23] appear to be somewhat separate. Secondarily, we used electromyography (EMG) to evaluate alterations in β-band intermuscular coherence obtained from muscles involved in the performance of the motor skill task. Intermuscular coherence is a measure of the similarity of a pair of EMG signals in the frequency domain [25]. Coherence between muscles is typically found in two frequency bands, α (5–15 Hz) and β (15–35 Hz). Sharing similarities and commonly considered to be underpinned by similar mechanisms as corticomuscular coherence, β-band intermuscular coherence is often used as an indirect measure of corticospinal activity that reflects common rhythmic drive from M1 to the assessed muscles [26,27,28,29]. Assessing intermuscular coherence in the current study provided a window into neurophysiological changes that may support exercise and practice schedule effects on motor skill consolidation.

## 2. Materials and Methods

### 2.1. Overview of Experimental Design

**Figure 1:**
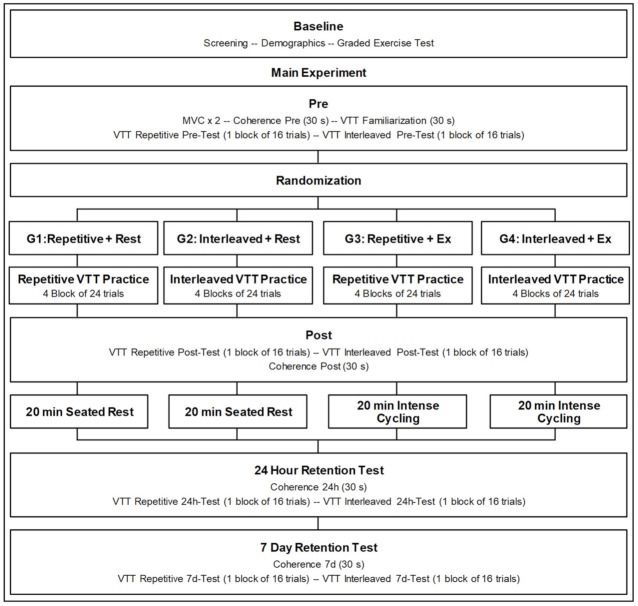
Overview of study design. MVC, maximum voluntary contraction; VTT, visuomotor tracking task; G1–G4, Groups 1–4; 24 h, 24 h; 7d, 7-day.

Participants in the study completed one baseline and three experimental sessions. During the baseline session, demographic information was recorded, and a graded maximal exercise test was performed. Experimental sessions started between 48 h and 92 days (median = 10 days) following the baseline session, were performed at the same time of day for each participant (±2 h), and involved (1) visuomotor tracking task (VTT) practice followed by a 20 min period of rest or a 20 min bout of high-intensity interval lower-limb cycling, (2) a 24 h retention test, and (3) a 7-day retention test. Participants were allocated using stratified randomization based on age, sex, and test performance in the first experimental session to one of four groups that, in the first experimental session, performed either (1) VTT practice with a repetitive schedule followed by rest (REP + REST), (2) VTT practice with an interleaved schedule followed by rest (INL + REST), (3) VTT practice with a repetitive schedule followed by exercise (REP + EX), or (4) VTT practice with an interleaved schedule followed by exercise (INL + EX). Retention sessions were conducted in the same manner across groups. Intermuscular coherence was measured before and after VTT practice and immediately following retention tests. An overview of the experimental design is presented in Figure 1.

### 2.2. Participants

Forty-eight young, healthy, right-handed adults (36M, 12F; mean age: 21.7 ± 4.0 years) participated. Participants completed the Physical Activity Readiness Questionnaire (PAR-Q) and were screened to ensure that exercise could be completed safely based on resting heart rate and blood pressure. Participants were considered not eligible if they had a history of neurological or psychiatric diseases, any health condition known to be aggravated by high-intensity exercise, or vision or hearing impairments. Participants were instructed to refrain from exercise for four hours before and after all laboratory visits. Participants were also instructed to refrain from heavy meals before the exercise test, caffeine on the morning of any sessions, as well as smoking or alcohol before any sessions. The study was approved by the University of Regina ethics review board for research with human participants (file #2019-069).

### 2.3. Graded Exercise Test

In the baseline session, participants completed a graded maximal exercise test on a cycle ergometer to determine cardiorespiratory fitness through the measurement of maximal oxygen uptake (VO_2_ peak). The exercise test was performed on a stationary cycle ergometer (Ergoselect 200, Ergoline, Germany). The test began with a five-minute warmup at a fixed workload of 50 W and a self-selected cadence. After the warmup, the cycle was set to 100 W for men or 50 W for women and the workload gradually increased by 30 W increments every 30 s. Participants were instructed to maintain a cadence of 90 revolutions per minute (rpm) until exhaustion, defined as the point at which participants could no longer maintain a minimum cycling cadence of 70 rpm. A metabolic cart (TrueOne 2400, ParvoMedics, Salt Lake City, UT, USA) was used to measure expired O_2_ and CO_2_ concentrations and airflow. Heart rate was measured throughout the duration of the test with a three-lead electrocardiograph (ECG) collected using LabChart software (LabChart 8.0, AD Instruments, Colorado Springs, CO, USA). The rating of perceived exertion was verbally reported using the Borg scale (6–20) after the warmup period and in one-minute increments throughout the graded exercise test.

### 2.4. Questionnaires

In the baseline session, participants also completed the shortened International Physical Activity Questionnaire (IPAQ), which determined the activity levels for the last 7 days for the participant. After each experimental session, the Stanford Sleepiness Scale and visual analog scales that measured the levels of attention and effort dedicated to the performance of the VTT were completed. Participants also provided information on their typical number of hours slept in a night and the number of hours slept in the night prior to each experimental session.

### 2.5. Maximum Voluntary Contractions (MVCs)

At the outset of the first experimental session, the force generated during a maximum voluntary pinch grip contraction (MVC) was measured to determine force target levels to be used during the measurement of intermuscular coherence and VTT. With forearms resting on a table, participants held a pinch grip force transducer between the thumb and index finger of the dominant hand. The force transducer consisted of a load cell (UU2 S-type, Dacell, Korea) with custom 3D-printed finger grips attached. The load cell signal was amplified by 400× (DN-AM110, Dacell, Korea), low-pass filtered at 10 Hz, and sampled at 100 Hz with a data-acquisition device (USB-6002, National Instruments, Austin, TX, USA). Two MVCs lasting 5 s in duration and separated by approximately three minutes from the previous maximal effort were performed. Participants were provided with visual feedback of their force production and received verbal encouragement to perform maximally. MVC force was derived over a 500 ms interval centered on the region of maximal force production during the contraction. The highest value across the two trials was considered to represent the MVC, which was then set to 20% for the VTT.

### 2.6. Intermuscular Coherence

Coherence in activity across the abductor pollicis brevis (APB) and first dorsal interosseous (FDI) muscles of the dominant hand was determined from a 30 s sub-maximal (i.e., 10% MVC force) pinch grip contraction performed before (pre) and after (post) VTT practice in the first experimental session and at the end of each retention session (see Figure 1). Surface EMG was recorded using 1 cm × 1 cm square electrodes (Tenby Medical, Vulcan, AB, Canada) placed in a bipolar configuration over the APB and FDI muscles of the dominant hand. EMG signals were collected using LabChart software (LabChart 8.0, AD Instruments, Colorado Springs, CO, USA) and were pre-amplified (1000×) and band-pass filtered at 10–1000 Hz with PowerLab amplification and EMG systems (AD Instruments, Colorado Springs, CO, USA). EMG data were recorded continuously over coherence trials with a sampling frequency of 2000 Hz. During the 30 s trial, participants were given verbal encouragement and visual feedback to maintain a consistent level of force. Intermuscular coherence was calculated based on the middle 20 s of the 30 s trials, using scripts based on those developed by [30]. Segments with lengths totaling 1024 were used, giving 39 segments per recording with a spectral resolution of 1.95 Hz. A 95% confidence limit was calculated for the coherence spectra using previously developed equations [30]. All intermuscular coherence data were visually inspected for signs of crosstalk, such as high coherence across a wide range of frequencies and near-zero lag synchronization in the time domain as determined by cumulant density plots [31]. No data displaying these characteristics were observed. The mean level of coherence in the β-frequency band (15–35 Hz) was considered representative of common corticomuscular drive [26]. The mean β-coherence on the pre- and post-tests, a change in β-coherence over the acquisition period (i.e., post-to-pre ratio), and a change in β-coherence over the consolidation periods (i.e., 24 h-to-post ratio, 7 d-to-post ratio) were considered in statistical analyses described below.

### 2.7. Visuomotor Tracking Task (VTT)

The VTT used in this study was custom-programmed (Python, Python Software Foundation, Wilmington, DE, USA) and adapted from a tracking task used to evaluate changes in corticospinal excitability with motor skill learning [32,33,34] and exercise effects on motor skill learning in prior work [8,35]. Participants were seated in front of a 27-inch computer monitor using a modified office chair that restricted movement with their forearms resting on the table in front of them, their wrists in a neutral position, and with a force transducer held between their thumb and index finger on their dominant hand (see MVC subsection above for specifications). To maintain consistency, participants were given verbal, scripted instructions. When performing the VTT, participants used pinch grip force to control the vertical position of a cursor that was moving across a computer monitor at a constant horizontal velocity. In a given trial, a set of targets (i.e., red rectangles) requiring different pinch grip force levels was presented, with the top of the screen corresponding to 20% of an individual’s maximum pinch grip force determined from the pre-test MVC. Participants were instructed to manipulate their pinch grip force to keep the cursor within the red rectangular targets presented in each trial. VTT performance was quantified as a number between 0 and 100 representing the Time on Target (ToT%) as a percentage of the trial duration.

#### 2.7.1. VTT Familiarization and Tests

**Figure 2:**
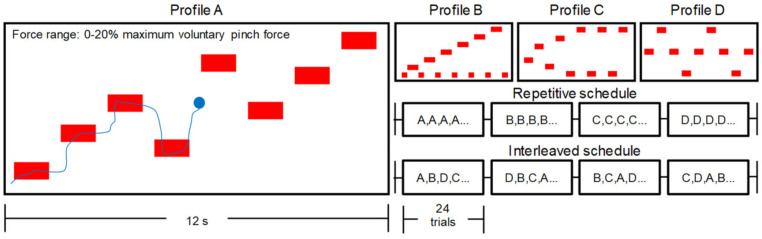
Depiction of visuomotor tracking task (VTT). Profiles A–D represent the four different target profiles that were used in the study.

Before the pre-tests (see Figure 1), all participants were familiarized to the VTT by performing a 30-s trial in which the cursor was controlled by the pinch grip force transducer without any force targets presented. During this trial, participants were instructed to squeeze the force transducer to move the cursor up and down as it traveled horizontally across the screen. Next, participants completed tests of VTT performance with repetitive and then interleaved schedules of four different force target profiles (see Figure 2, profiles A–D). The force target profiles of the four trials were the same across repetitive and interleaved tests, did not change across the experimental sessions, and were also used in VTT practice, as described below. Each test consisted of one set of 16 trials (12 s per trial), with the repetitive test presenting four of each force target profile in a row and the interleaved test presenting four of each force target profile organized in random order. In the interleaved test, the same force target profile was never presented twice in a row. The four target profiles were tested in the same order for each participant. Testing all participants under both repetitive and interleaved schedules ensured the evaluation of practice-test compatibility across groups [19,36,37,38]. Participants viewed the cursor and the target profiles while performing the tests but did not receive any augmented feedback. The tests were repeated after practice in the first experimental session and in each retention session. ToT% on the pre- and post-tests, the change in ToT% over the acquisition period (i.e., post-to-pre ratio), and the change in ToT% over the consolidation periods (i.e., 24 h-to-post ratio, 7 d-to-post ratio) were considered in statistical analyses described below.

#### 2.7.2. VTT Practice

VTT practice in the first experimental session consisted of four sets of 24 trials (12 s per trial) for just under 20 min of practice. Participants allocated to groups performing repetitive practice (i.e., REP + REST, REP + EX) performed each of the four force target profiles in a separate set. Specifically, a set consisting of 24 trials of only force target profile A was completed before moving on to a set of profile B, then profile C, then profile D. For participants allocated to groups performing interleaved practice (i.e., INL + REST, INL + EX), the four force target profiles were presented in a random order across all four sets (i.e., each set of practice consisted of a random mix of all four force target profiles). The only restrictions on randomization were that each set consisted of six trials of each force target profile and that the same force target profile was never presented twice in a row. During practice, participants received augmented feedback in the form of their ToT% result presented on the screen after each trial and their average ToT% shown at the halfway point and at the end of each set. All participants were repeatedly encouraged to ‘do their best’ and improve performance throughout practice.

### 2.8. Standardized Acute Exercise Bout

In the first experimental session, participants allocated to the exercise groups (i.e., REP + EX, INL + EX) completed a standardized acute cardiorespiratory exercise bout at the end of the session (i.e., after VTT practice and all tests). The protocol for the standardized exercise bout was similar to previous work [1,4,8,35,39]. Briefly, participants performed a 5 min warmup at 50 watts at a self-selected cadence, followed by three sets of high-intensity cycling intervals for three minutes set at 90% of the maximum workload from the baseline exercise test. Between intervals, participants performed two minutes of low-intensity cycling at 50 watts at a self-selected cadence. The rating of perceived exertion (RPE) was verbally reported using the Borg scale (6–20) after each three-minute exercise interval. In contrast, participants allocated to the rest groups (i.e., REP + REST, INL + REST) were seated for 20 min and engaged in light conversation at the end of the session. Heartrate data (ECG) and APB and FDI muscle activity (EMG) were continuously monitored using LabChart software (LabChart 8.0, AD Instruments, Colorado Springs, CO, USA) throughout the exercise and rest periods, and participants were instructed to keep their hands as relaxed as possible. The purpose of monitoring the ECG data was to ensure that the workload for the exercise bout elicited a heartrate response consistent with high-intensity cycling and to ensure that participants in rest groups maintained a resting heartrate during the rest periods. Monitoring EMG data allowed the experimenters to ensure that the hand muscles engaged in the pinch grip task were minimally active during exercise and rest periods.

### 2.9. Statistical Analyses

#### 2.9.1. Normality

The normality of all data included in statistical tests was first examined through visual inspection of skewness and kurtosis and objective testing of normality with the Shapiro–Wilk test with a significance level set at *p* < 0.001 [40]. Continuous variables that were identified as non-normal were log-transformed for further analyses, including pre-coherence, post-coherence, the post-to-pre coherence ratio, the 24h-to-post coherence ratio, and the 7d-to-post coherence ratio.

#### 2.9.2. Participant Characteristics and Questionnaires

Age, body mass index (BMI), VO_2_ peak, W_max_, hours slept before each session, and the difference in hours slept to a typical night’s sleep at each session were analyzed with separate two-way practice schedule (i.e., repetitive versus interleaved) by condition (i.e., rest versus exercise) analyses of variance (ANOVAs). Stanford Sleepiness Scale and visual analog scale data at each time point were analyzed, first with Mann–Whitney U tests comparing the repetitive versus interleaved groups and the rest versus exercise groups, and second with a Kruskal–Wallis test comparing all four groups. Chi-squared tests determined whether there were any differences between groups in the frequency of males and females and the frequency of those presenting with high, moderate, and low levels of physical activity on the IPAQ.

#### 2.9.3. VTT Practice Session

Practice-schedule-by-condition ANOVAs compared pre-ToT%, post-ToT%, and post-to-pre ToT% ratios (i.e., change in performance) for repetitive and interleaved tests separately. Potential differences in pre-coherence (log-transformed), post-coherence (log-transformed), and the post-to-pre coherence ratio (i.e., change in coherence, log-transformed) were also evaluated by separate practice-schedule-by-condition ANOVAs.

#### 2.9.4. Retention Sessions

Primary analyses focused on changes in VTT performance (i.e., ToT%) and coherence over the consolidation periods. As such, the dependent variables in six separate two-way practice schedule (repetitive versus interleaved) by condition (rest versus exercise) ANOVAs were the 24 h-to-post and 7 d-to-post ratios for ToT% on repetitive tests, ToT% on interleaved tests, and coherence (log-transformed). Post-hoc Bonferroni-corrected *t*-tests were conducted to further examine significant interactions by comparing the rest and exercise groups separately for those who completed repetitive practice and those who completed interleaved practice.

#### 2.9.5. Sample Size Calculation

The sample size calculation focused on the primary hypothesis that an interleaved practice schedule and post-practice exercise would demonstrate an additive benefit for motor skill consolidation. Based on prior work that reported a large effect of interleaved practice relative to repetitive practice on motor skill consolidation at a 24 h retention test (Cohen’s d estimated from Figure 2 as ~1.50) [23], as well as a large effect of post-practice exercise relative to rest on motor skill consolidation at 24 h and 7-day retention tests (Cohen’s d ranged from 1.26–1.70 across specific comparisons) [35], the expected effect size was conservatively set at Cohen’s d = 1.26 for each main effect (i.e., practice schedule and condition). Thus, with β = 0.80 and α = 0.05, we determined that approximately *n* = 11 participants per group would allow for testing of the primary hypothesis. We recruited a total of 11–13 participants per group to accommodate for potential drop-out or attrition.

#### 2.9.6. Reporting

The significance level was set to *p* < 0.05 for all statistical tests, besides Bonferroni-corrected post-hoc *t*-tests, which used a significance of *p* < 0.025. Effect sizes are reported as partial eta squared (η^2^*p*) for ANOVAs, eta squared (η^2^) for Mann–Whitney U and Kruskal–Wallis tests, phi (ϕ) for Chi-squared tests, and Cohen’s d for *t*-tests. Statistical analyses were conducted using SPSS software (v 25.0, IBM Corporation, New York, NY, USA).

## 3. Results

### 3.1. Participant Characteristics and Questionnaires

There were no significant main effects or interactions when considering ANOVAs examining age, BMI, VO_2_ peak, W_max_, hours slept before each session, and the difference in hours slept to a typical night’s sleep (*p* range: 0.08–0.97, η^2^*p* range: <0.001–0.08). Non-parametric tests also indicated no significant difference at any time point between experimental groups in the Stanford Sleepiness Questionnaire, and visual analog scale ratings of attention and effort (*p* range: 0.11–0.97, η^2^ range: <0.001–0.06). Likewise, Chi-squared tests indicated no significant differences in sex or physical activity levels between groups (*p* range: 0.07–1.00, ϕ range: −0.27–0.00); however, there was a trend for fewer females to be allocated to the exercise groups (χ^2^_(1)_ = 3.37, *p* = 0.07, ϕ = −0.27). Table 1 and Table 2 provide an overview of these measures across groups.

### 3.2. Practice Session

ANOVAs identified no significant main effects or interactions when considering pre- and post-ToT% and post-to-pre-ToT% ratios for both repetitive and interleaved tests, as well as for pre- and post-coherence (log-transformed), and the post-to-pre-coherence ratios (log-transformed) (*p* range: 0.11–0.99, η^2^*p* range: <0.001–0.06). The means and standard deviations of these variables for each group are presented in Table 1.

### 3.3. Retention Sessions

**Figure 3:**
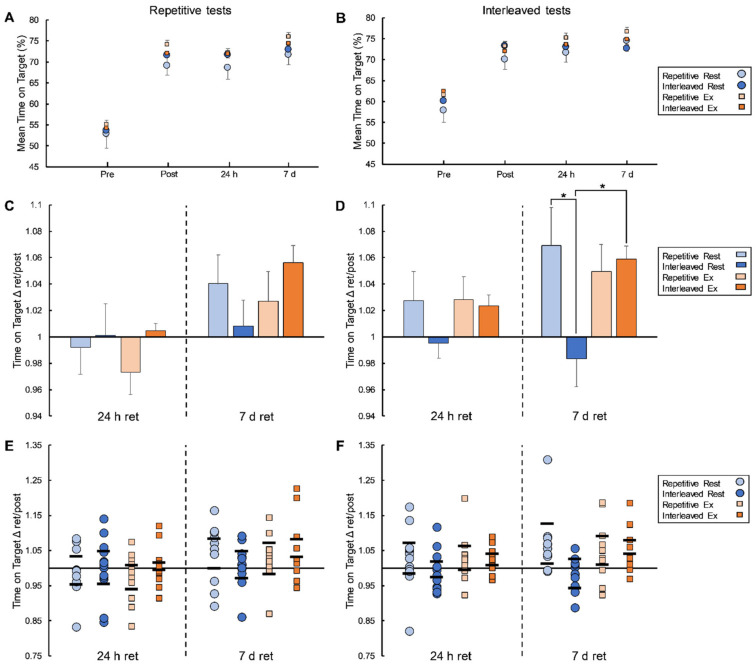
Visuomotor tracking task (VTT) results. Panels (**A**,**B**) depict raw time on target (ToT%) data for repetitive (**A**) and interleaved (**B**) tests when averaged across participants in each group for each time point. Panels (**C**,**D**) show average change in ToT% for repetitive (**C**) and interleaved tests (**D**) over the consolidation periods for each group, with performance at each retention test expressed as a ratio relative to post-practice test performance. Error bars in panels (**A**–**D**) reflect standard error. Asterisks (*) in panels (**C**,**D**) denote statistically significant differences between groups. Panels (**E**,**F**) show individual data points contributing to the group averages in (**C**,**D**). Horizontal lines in panels (**E**,**F**) represent 95% confidence intervals.

No significant main effects or interactions in the 24h-to-post ratios for repetitive or interleaved test ToT% or coherence (log transformed) were detected (all *p*-values > 0.32, η^2^*p*-values < 0.03). There also were no significant main effects or interactions in the 7d-to-post ToT% ratios for the repetitive test on the VTT (all *p*-values > 0.15, η^2^*p*-values < 0.05). However, considering the 7d-to-post ToT% ratio for the interleaved test on the VTT, there was a trend for a main effect of practice schedule (F_(1,44)_ = 3.70, *p* = 0.06, η^2^*p* = 0.08) and a significant practice-schedule-by-condition interaction (F_(1,44)_ = 5.70, *p* = 0.02, η^2^*p* = 0.12). Post-hoc *t*-tests indicated that the 7d-to-post ToT% ratio for the interleaved test was higher for the exercise group compared to the rest group among those who completed interleaved practice (t_(22)_ = −3.40, *p* = 0.003, d = −1.37), but not among those who completed repetitive practice (t_(22)_ = 0.59, *p* = 0.56, d = 0.24). Additionally, the 7d-to-post ToT% ratio for the interleaved test was higher in those who practiced with a repetitive schedule compared to those who practiced with an interleaved schedule among the rest groups (t_(23)_ = 3.32, *p* = 0.003, d = 1.31) but not the exercise groups (t_(21)_ = −0.30, *p* = 0.77, d = −0.13) (see Figure 3). 

**Figure 4:**
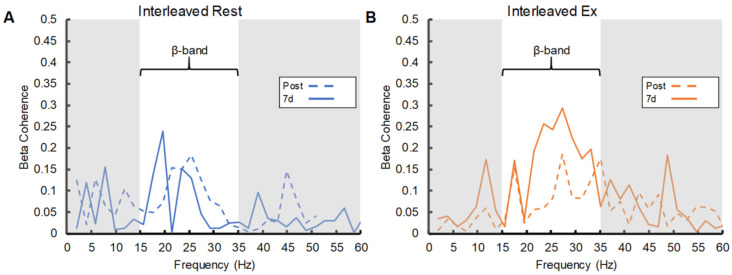
Intermuscular coherence data from representative participants and time points. Panel (**A**) shows data obtained from an individual in the interleaved practice and rest group. Panel (**B**) depicts data obtained from an individual in the interleaved practice and exercise group.

**Figure 5:**
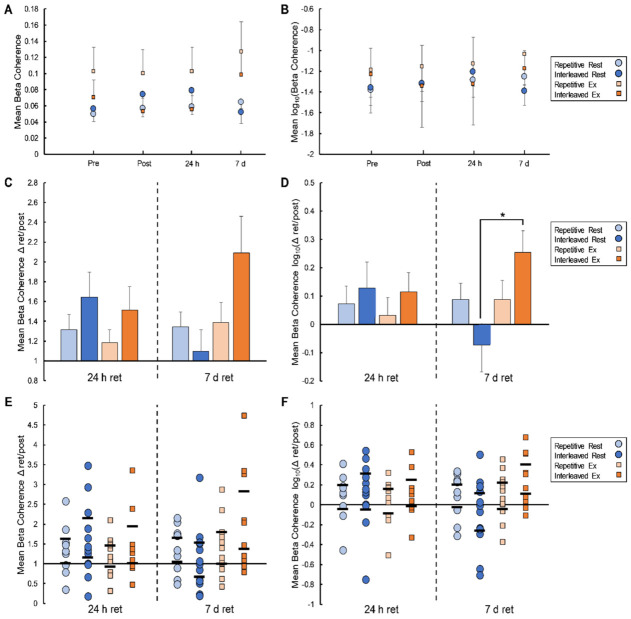
Intermuscular coherence results. Panels (**A**,**B**) depict raw (**A**) and log-transformed (**B**) intermuscular beta coherence data when averaged across participants in each group for each time point. Panels (**C**,**D**) show average change in mean beta coherence (**C**) and log-transformed mean beta coherence (**D**) over the consolidation periods for each group, with coherence at each retention test expressed as a ratio relative to post-practice coherence. Error bars in panels (**A**–**D**) reflect standard error. Asterisks (*) in panels (**C**,**D**) denote statistically significant differences between groups. Panels (**E**,**F**) show individual data points contributing to the group averages in (**C**,**D**). Horizontal lines in panels (**E**,**F**) represent 95% confidence intervals.

There was also a significant main effect of condition (F_(1,44)_ = 4.48, *p* = 0.04, η^2^*p* = 0.09) and a significant practice-schedule-by-condition interaction (F_(1,44)_ = 4.46, *p* = 0.04, η^2^*p* = 0.09) in the 7d-to-post coherence ratio. Similar to the ToT% results, post-hoc tests indicated that exercise enhanced the 7d-to-post coherence ratio for those who completed interleaved practice (t_(22)_ = −2.61, *p* = 0.02, d = −1.53) but not for those who completed repetitive practice (t_(22)_ = −0.01, *p* = 0.99, d < 0.01). Representative intermuscular coherence data from single participants who completed interleaved practice followed by rest and interleaved practice followed by exercise are presented in Figure 4 panels A and B, respectively. Figure 5 provides a summary of the intermuscular coherence results across all study groups.

## 4. Discussion

We tested the hypothesis that coupling an interleaved practice schedule with a post-practice bout of cardiorespiratory exercise would have an additive benefit for motor skill consolidation. To gain insight on how changes in the corticospinal system might contribute to behavioral effects, the common M1 drive to muscles involved in skill performance was determined using β-band intermuscular coherence. Our hypothesis was not supported, as the interleaved practice schedule did not enhance motor skill consolidation in this work. Instead, results obtained at the 7-day retention time point suggested that exercise promoted skill consolidation when performed after interleaved skill practice only, with no exercise effect on skill consolidation when performed after repetitive skill practice. Intermuscular coherence measures mirrored the behavioral observations, with a greater increase in β-band coherence at the 7-day retention time point among those who exercised following interleaved practice relative to those who rested following interleaved practice.

### 4.1. Interleaved Practice Did Not Enhance Motor Skill Consolidation

Contextual interference is a topic of great interest in the motor skill learning literature [17]. Generally, when studying contextual interference, the expected observation is that, relative to a repetitive practice schedule, an interleaved practice schedule results in a slower rate of skill acquisition and/or lesser pre-to-post-practice change in performance but greater long-term retention and offline gains assessed >24 h following practice [19]. Such findings are known to extend across testing contexts, such that the benefits of interleaved practice may be observed in a retention test administered in either a repetitive or interleaved format [41]. Findings from the current study did not align with this expected contextual interference effect. First, there was no evidence of an effect of practice schedule on the pre-to-post practice change in performance (i.e., within-session) or off-line change at the 24 h retention test. Second, results from the rest groups at the 7-day retention test suggest that the repetitive practice schedule promoted off-line gains to a greater degree than the interleaved practice schedule. This difference was apparent for both the repetitive (Figure 3C) and interleaved (Figure 3D) test formats, but only statistically significant for the tests delivered with the interleaved format.

Findings that contradict the beneficial effect of contextual interference and demonstrate a learning benefit associated with repetitive practice have been reported elsewhere [42,43,44,45,46]. A common feature of these prior studies was that they employed tasks with relative force or relative timing demands (e.g., a key-press sequence with specific timing goals for each key press [45]), rather than a simple goal movement outcome as was used in many of the early contextual interference studies (e.g., knocking down barriers in a prescribed order as fast as possible [19]). It has been posited that tasks with such “relative” demands increase the complexity of the task and the associated processing load, which then alters the contextual interference effect [47,48]. While the learning of simple tasks is enhanced by a practice schedule that increases the demands on memory (i.e., interleaved schedule), complex task learning may be benefited more by practice schedules that reduce the demands on memory (i.e., repetitive schedule) [48]. In the current work, the task (i.e., VTT) was adapted from prior studies that demonstrated acute exercise benefits for motor learning [1,3,6,8,35]. Notably, the task placed both relative force and relative timing demands on the learners and, as such, would be considered a complex task as it relates to other contextual interference literature [47,48]. Thus, the complexity of the task used here was likely not amenable to demonstrating the classic contextual interference effect and instead yielded a learning benefit for the repetitive practice schedule among the rest groups.

### 4.2. Exercise Enhanced Motor Skill Consolidation following Interleaved Practice Only

While, in retrospect, the task utilized was not well-suited to demonstrate a beneficial contextual interference effect, our experimental design still allowed us to observe an interaction between memory demands imposed by different practice schedules and the subsequent effects of cardiorespiratory exercise on motor skill consolidation. Results demonstrated a benefit for post-practice exercise on motor skill consolidation at the 7-day retention time point only among participants who performed interleaved practice. Given that repetitive practice benefited motor skill consolidation relative to interleaved practice in the rest groups at the 7-day retention time point, another interpretation might be that the interleaved practice schedule impaired motor skill consolidation relative to repetitive practice, with the post-practice exercise exerting a protective effect (see Figure 3D,F).

Prior studies using short-term [49] and long-term [50] retention intervals demonstrated that an acute bout of cardiorespiratory exercise following practice of a motor sequence task can protect against the negative effects of exposure to an interfering motor sequence task. Other work demonstrated that acute cardiorespiratory exercise following motor skill practice also protects consolidation from interference produced by a declarative learning task [51] and from a non-invasive brain stimulation protocol designed to suppress corticospinal excitability [52]. Together, these findings suggest that post-practice exercise may optimize the use of neural resources involved in skill consolidation to overcome potentially detrimental effects of memory interference [51]. Given that interleaved practice schedules are designed to provide contextual interference within the learning process [17], our current findings extend this prior work to further suggest that acute cardiorespiratory exercise benefits for motor learning are underpinned by an influence on memory interference. While the rest group that performed interleaved practice experienced a degree of interference that was detrimental to learning the task, the introduction of post-practice exercise appears to have mitigated these negative interference effects to preserve off-line gains reflective of motor skill consolidation. Consistent with prior work [8,35], the impact of exercise was most evident at the long-term 7-day retention time point, suggesting a critical relationship between exercise and sleep on motor skill consolidation. Generally, the literature on contextual interference that considers task complexity suggests that complex skills may require initial practice with a repetitive schedule to reduce memory demands until a certain level of task proficiency is achieved, after which interleaved practice scheduling might be introduced [48]. Although speculative and dependent on our consideration of the current motor skill as “complex”, current findings suggest that post-practice exercise may provide an approach to accelerate the progression of complex motor skill practice towards the use of interleaved scheduling without negatively impacting motor skill consolidation.

### 4.3. Exercise Benefits for Motor Skill Consolidation Were Accompanied by Increased Intermuscular Coherence

β-band intermuscular coherence between the APB and FDI muscles was increased at the 7-day retention relative to the post-practice time point to a greater degree for those who exercised compared to those who rested following interleaved practice (Figure 4 and Figure 5). Changes in β-band intermuscular coherence with motor learning are not well-documented in the literature; however, some information may be drawn from studies of corticomuscular (i.e., electroencephalography-EMG) and intramuscular (i.e., EMG-EMG) coherence based on the notion of similar neurophysiological underpinnings [25,26,27,28]. For example, Perez et al. [53] reported an increase in both corticomuscular and intramuscular coherence within the β-frequency band for the tibialis anterior muscle immediately following practice of a lower-limb visuomotor tracking task. Altered coherence was tied to motor learning, as no changes were observed following a control session that involved similar levels of muscular contraction without a learning component [53]. A more recent study evaluated changes in β-band coherence, both corticomuscular and intramuscular, between the end of motor skill practice and 24 h and 7-day retention tests [54]. In this study, transcranial alternating current stimulation (tACS) or sham stimulation was delivered immediately after practice of a lower-limb visuomotor tracking task that again involved dorsiflexion movements to test potential effects on post-practice skill consolidation. Results demonstrated a significant increase in β-band corticomuscular and intramuscular coherence from post-practice to both retention tests in the tACS group relative to sham, with changes in corticomuscular coherence that were positively correlated with task learning [54]. These findings were taken to suggest that tACS-facilitated overnight increases in β-band coherence along the corticospinal pathway may support post-practice skill consolidation.

Results from the current study further support the idea that post-practice changes in oscillatory coupling within the corticospinal system are supportive of motor skill consolidation. Presently, these changes were measured via β-band intermuscular coherence between two muscles used in concert to perform a motor skill task, with evidence that post-practice exercise facilitated the off-line coupling that occurred. As with the behavioral results, the cardiorespiratory exercise effect was only evident at the 7-day retention time point for groups who practiced the task with an interleaved schedule. Prior work has demonstrated that changes in β-band intermuscular coherence between upper limb muscles following the delivery of transcranial direct current stimulation reflect changes in corticospinal excitability assessed via motor-evoked potentials elicited by single-pulse transcranial magnetic stimulation for the same muscles [55]. Thus, changes in coherence observed here may align with the finding that cardiorespiratory exercise facilitated post-practice increases in corticospinal excitability involved in motor learning [24], while advancing our understanding of changes in the corticospinal system that may continue to evolve through overnight consolidation processes. The emergence of the exercise effect on intermuscular coherence only among those who performed interleaved practice also suggests some interaction with the challenge provided, or memory and cognitive demands required, during practice. Yet, prior evidence of greater involvement of M1 in motor skill consolidation following repetitive compared to interleaved practice [23] raises questions. Although speculative, it may be that the intermuscular coherence results reflect exercise-induced facilitation of the characteristic shift in brain activity towards M1 as a skill is learned [22], and that the learning process triggered by interleaved practice provided greater opportunity for detection of this effect.

### 4.4. Limitations

There are several limitations to the study design and measurement techniques that may influence the interpretation of the current findings. The sample size and magnitude of the reported effects were modest, but similar to related work (e.g., [1,2,3,4,5,6]). Given that the experimental control for the bout of high-intensity exercise was a matched duration of seated rest, it is possible that a negative impact of the rest contributed to the differences observed. Another consideration relates to the potential influence of gripping of the handlebars on the cycle ergometer during the exercise bout, a limitation common in related work examining exercise effects on corticospinal excitability and motor learning (e.g., [1,8,13,24]); however, hand muscle activity (i.e., EMG) was monitored continuously during the exercise sessions, and verbal feedback was given to encourage minimal contraction. Considering the measurement of intermuscular coherence, the proximity of the muscles of interest introduced the risk of crosstalk contributing to coherence values [56,57]. To mitigate these potential contributions, data were screened for crosstalk via accepted approaches used in prior work (i.e., the inspection of values across a range of frequency bands, the consideration of time lag synchronization in cumulant density plots) [31,58,59]. Further, while we carefully landmarked electrode locations across sessions, it should be noted that slight differences in EMG recording sites likely introduced variability in intermuscular coherence measurements across skill practice and retention study time points. Finally, while pinch grip force and background EMG was continuously monitored during the coherence measurements to ensure measurements were comparable across time points, it is possible, although unlikely [53], that coherence values were influenced by slight fluctuations or differences in contraction levels.

## 5. Conclusions

Under the present experimental conditions, a bout of high-intensity cycling after motor skill practice with an interleaved, but not repetitive, practice schedule was beneficial for off-line motor skill consolidation. Changes in β-band intermuscular coherence that corresponded with behavioral measures suggest that exercise effects on motor skill consolidation may be partly underpinned by an impact on oscillatory activity within the corticospinal system.

## Figures and Tables

**Table 1:** Participant characteristics.

	REP + REST	INL + REST	REP + EX	INL + EX
N	12	13	12	11
Age (years)	23.2 ± 4.9	20.8 ± 2.8	23.1 ± 4.4	22.7 ± 5.5
Sex (M/F)	8/4	8/5	10/2	10/1
BMI (kg/m^2^)	23.8 ± 4.4	22.2 ± 3.4	24.3 ± 3.3	26.3 ± 5.2
VO_2_ peak (mL/min/kg) *	37.4 ± 1.8	35.8 ± 7.8	37.2 ± 9.3	42.1 ± 8.1
IPAQ score (H/M/L)	8/2/2	8/1/4	8/2/2	7/3/1
*W*_max_ (W)	175.5 ± 50.0	181.5 ± 56.8	183.3 ± 34.5	215.5 ± 55.7
VTT Repetitive Pre-ToT%	52.9 ± 12.1	53.6 ± 8.2	55.1 ± 8.0	54.3 ± 7.2
VTT Repetitive Post-ToT%	69.1 ± 7.7	71.6 ± 5.8	74.1 ± 6.2	72.1 ± 8.2
VTT Repetitive Post-to-Pre ToT% ratio	1.36 ± 0.26	1.35 ± 0.16	1.36 ± 0.13	1.34 ± 0.16
VTT Interleaved Pre-ToT%	57.8 ± 9.7	60.2 ± 7.7	61.6 ± 11.6	62.5 ± 6.7
VTT Interleaved Post-ToT%	70.0 ± 8.3	73.3 ± 5.9	73.2 ± 6.2	72.0 ± 7.0
VTT Interleaved Post-to-Pre ToT% ratio	1.23 ± 0.2	1.23 ± 0.12	1.22 ± 0.23	1.16 ± 0.11
Coh Pre-A_x_ (0–1)	0.05 ± 0.03	0.06 ± 0.04	0.10 ± 0.11	0.07 ± 0.04
Coh Post-A_x_ (0–1)	0.06 ± 0.04	0.07 ± 0.07	0.10 ± 0.08	0.05 ± 0.03
Coh Post-to-Pre ratio	1.25 ± 0.52	1.75 ± 2.29	1.34 ± 0.95	0.83 ± 0.26

* VO_2_ peak data were not available for one participant in the REP + REST group, two participants in the REP + EX group, and one participant in the INL + EX group as a result of COVID-19-related research restrictions that precluded measurement of expired gases. For standardized exercise bouts, these individuals were assigned workloads consistent with the average used for males or females in prior study participants. The workloads were then adjusted in real time to obtain a rating of perceived exertion between “Very hard” and “Extremely hard” (i.e., 17–19/20). REP + REST, repetitive schedule followed by rest; INL + REST, interleaved schedule followed by rest; REP + EX, repetitive schedule followed by exercise; INL + EX, interleaved schedule followed by exercise; M, male; F, female; VO_2_, volume of oxygen consumption; IPAQ, International Physical Activity Questionnaire; *W*_max_, maximum power output from graded exercise test; ToT, time on target; Coh, coherence. Results are presented as mean ± SD.

**Table 2:** Participant reports of sleep, sleepiness, attention, and effort.

	REP + REST	INL + REST	REP + EX	INL + EX
Hours slept prior to:				
(i) VTT Practice	6.4 ± 1.4	6.9 ± 1.4	7.2 ± 1.0	6.2 ± 1.8
(ii) 24 h Ret	7.3 ± 1.7	6.8 ± 1.4	7.0 ± 1.3	7.7 ± 1.4
(iii) 7 d Ret	7.1 ± 1.5	6.7 ± 2.2	7.4 ± 1.8	7.4 ± 1.0
Difference from typical night’s sleep:				
(i) VTT Practice	−1.1 ± 1.7	−0.2 ± 1.3	−0.1 ± 0.8	−0.9 ± 1.8
(ii) 24 h Ret	−0.2 ± 1.9	−0.3 ± 1.1	−0.5 ± 1.0	0.8 ± 1.4
(iii) 7 d Ret	−0.4 ± 1.0	−0.4 ± 1.9	0.1 ± 1.4	0.3 ± 1.1
Stanford Sleepiness Scale:				
(i) VTT Practice	2.4 ± 1.4	3.0 ± 1.4	2.6 ± 1.4	3.3 ± 1.6
(ii) 24 h Ret	1.8 ± 0.8	2.2 ± 1.1	2.2 ± 0.9	2.5 ± 0.9
(iii) 7 d Ret	1.7 ± 0.7	2.2 ± 0.8	2.0 ± 0.7	2.1 ± 0.9
Attention VAS (0–10):				
(i) VTT Practice	7.5 ± 1.2	7.4 ± 1.4	7.8 ± 1.7	7.6 ± 1.8
(ii) 24 h Ret	8.9 ± 0.7	7.9 ± 2.5	8.5 ± 1.4	8.2 ± 1.1
(iii) 7 d Ret	8.8 ± 1.2	8.0 ± 2.1	8.7 ± 1.3	8.4 ± 1.2
Effort VAS (0–10):				
(i) VTT Practice	9.4 ± 0.6	8.9 ± 2.1	8.9 ± 1.1	8.9 ± 1.5
(ii) 24 h Ret	9.3 ± 0.8	8.6 ± 2.3	9.3 ± 0.7	9.2 ± 0.7
(iii) 7 d Ret	9.2 ± 1.1	9.2 ± 1.7	9.5 ± 0.6	8.9 ± 1.1

REP + REST, repetitive schedule followed by rest; INL + REST, interleaved schedule followed by rest; REP + EX, repetitive schedule followed by exercise; INL + EX, interleaved schedule followed by exercise; h, hour; d, day; Ret, retention; VAS, visual analog scale. Results are presented as mean ± SD.

## Data Availability

The data presented in this study are available upon reasonable request.
